# *Bordetella* Adenylate Cyclase-Hemolysin Toxins

**DOI:** 10.3390/toxins9090277

**Published:** 2017-09-11

**Authors:** Nicole Guiso

**Affiliations:** Institut Pasteur Unité de Prévention et Thérapies Moléculaires des Maladies Humaines, 25 rue du Dr. Roux, 75015 Paris, France; nicole.guiso@pasteur.fr; Tel.: +00-33-685-827-328

**Keywords:** *Bordetella* species, adenylate cyclase toxin, vaccine antigen

## Abstract

Adenylate cyclase-hemolysin toxin is secreted and produced by three classical species of the genus *Bordetella*: *Bordetella pertussis*, *B. parapertussis* and *B. bronchiseptica*. This toxin has several properties such as: (i) adenylate cyclase activity, enhanced after interaction with the eukaryotic protein, calmodulin; (ii) a pore-forming activity; (iii) an invasive activity. It plays an important role in the pathogenesis of these *Bordetella* species responsible for whooping cough in humans or persistent respiratory infections in mammals, by modulating host immune responses. In contrast with other *Bordetella* toxins or adhesins, lack of (or very low polymorphism) is observed in the structural gene encoding this toxin, supporting its importance as well as a potential role as a vaccine antigen against whooping cough. In this article, an overview of the investigations undertaken on this toxin is presented.

## 1. Introduction

The genus *Bordetella* comprises nine species. Among them, *B. pertussis*, *B. parapertussis* and *B. bronchiseptica* are so closely related, despite differences in host range, that they are often considered as subspecies of the same species and are called “classical species” [1]. *B. pertussis* is the agent of whooping cough, a severe respiratory disease in humans which can be dramatic for newborns and elderly subjects. *B. parapertussis* is the agent of respiratory disease in sheep and in humans; however, the clinical symptoms induced in humans last less than those induced by *B. pertussis* although clinically similar [2,3]. *B. bronchiseptica* is a respiratory pathogen for several mammal species and sometimes for immuno-suppressed patients [1]. *B. pertussis* and *B. parapertussis* appear to have independently emerged from a *B. bronchiseptica* ancestor through rearrangement and loss of genetic material [4,5]. Those three species are closely related and produce similar as well as specific virulence factors. The three species produce (i) similar adhesins such as filamentous hemagglutinin (FHA) and pertactin (PRN), but different fimbrial proteins (FIM); (ii) similar toxins such as adenylate cyclase-hemolysin (AC-Hly) toxin, tracheal cytotoxin and dermonecrotic toxin, but *B. pertussis* is the only species producing pertussis toxin (PT); (iii) *B. bronchiseptica* is the only species producing a flagellum, a functional *Bordetella* type-III secretion system effector A, and a type-VI secretion system [6,7,8]. In this review we will only focus on the properties of the AC-Hly toxin common to the three species.

## 2. *Bordetella pertussis* Adenylate Cyclase-Hemolysin Toxin

### 2.1. Structure

*B. pertussis* AC-Hly (Bp AC-Hly) was discovered in 1976 [9] and soon after shown to invade phagocytic cells and inhibit phagocytosis and oxidative burst in human neutrophils [10].

Since the sequencing of its structural gene, *cyaA* [11], it has been known that Bp AC-Hly toxin belongs to the Repeats in ToXins (RTX) family composed of toxins secreted by Gram-negative bacteria. These RTX toxins share several features such as (i) post-translational modification; (ii) C-terminal unprocessed secretion signal; (iii) export out of the cell by type I secretion systems (T1SS); and (iv) a C-terminal calcium-binding domain consisting of acidic glycine-rich nonapeptide repeats. Furthermore, most RTX toxins penetrate and permeabilize host cells. [12]. 

Bp AC-Hly is a 1706 amino acid protein containing several domains (i) an N-terminal adenylate cyclase domain of 364 amino acid residues; (ii) a translocation domain between residues 365 to 500; (iii) an hydrophobic domain between residues 500 to 750; (iv) a domain containing the two post-translational sites (Lys 860 and Lys 983) between residues 750 to 1000; and (v) a typical calcium-binding domain containing repeats of glycine and aspartate nona-peptides binding calcium, similar to all RTX toxins, between residues 1000 and 1706. This domain contains the receptor binding site and the C-terminal secretion system [13,14]. 

The post-translational modifications of Bp AC-Hly, first demonstrated by Barry et al. [15], are performed by an acyl-translational enzyme encoded by the *cyaC* gene. Acylation is essential for tight binding and interaction with human cells as well as for toxin activities [13]. Hackett et al. [16] showed that Bp AC-Hly is post-translationally modified by a palmitoylation on Lys 983. However, later it was found that the recombinant Bp AC-Hly toxin produced in *Escherichia coli* K-12, in presence of the cyaC protein, was also modified on Lys 860 [17]. Recently, we compared the post-translational modifications (PTM) of the Bp AC-Hly produced by the reference strain *B. pertussis* Tohama and by different recent *B. pertussis* clinical isolates. We showed that Bp AC-Hly harbors PTM at both sites: palmitoylations on Lysine 860 and palmoylations and myristoylations on Lysine 983 [18].

Three proteins, encoded by the *cyaBDE* genes of a Type I secretion system, are implicated in the secretion of the toxin. The secretion occurs directly from bacterial cytosol into the extracellular medium [12].

Bp AC-Hly interacts with two other virulence factors produced by *B. pertussis*, FHA [19] and PRN [20]. A direct physical interaction was characterized between AC-Hly toxin and FHA. This interaction may help to increase the local concentration of the toxin on the *B. pertussis* outer membrane. Adherence to host cells, mediated by FHA, may be coupled to AC-Hly toxin delivery [19]. These interactions might be of high importance for the conformation and maximal activity of the toxin. 

### 2.2. Regulation of Expression

The expression of this toxin is regulated by the two-component system called the BvgAS system [6], which also controls the expression of several other virulence factors of *B. pertussis*. The BvgAS system responds to environmental signals such as temperature, or biochemical signals such as nicotinic acid or sulfate, and allows the expression of the virulence factors. Experiments with mutants, deficient of BvgAS expression, demonstrated that this expression is necessary and sufficient for respiratory infection using a murine respiratory model [6].

However, expression of Bp AC-Hly was shown to be also regulated by other factors such as the anti-sigma factor RseA [21]. Recently, it was demonstrated that Rpo-E, a sigma factor which responds to environmental stresses, can indirectly modulate Bp AC-Hly, as well as PT, expression, through mechanisms under investigation [22]. It can be hypothesized that this is the basis of the increase in expression of AC-Hly by *B. pertussis* observed after collection of bacteria from the lungs of infected mice [23,24]. 

*B. pertussis* isolates, collected from human nasopharyngeal samples, can also produce more AC-Hly because they harbor a duplication of the *cya* locus [25]. These isolates are found to be not more virulent in the murine respiratory model or in a cellular model. However, this failure to demonstrate any difference could be due to the high instability of these isolates. It is possible that AC-Hly overexpression is necessary during human infection but not in vitro, or that it is unstable in vitro. Furthermore, the murine model may not be the best model for comparing clones producing different levels of AC-Hly. In fact, AC-Hly might have a higher affinity for the human receptor than for the murine receptor. It has also been shown that efficient secretion of AC-Hly requires an interaction with FHA [19]. As the isolate harboring a duplication of the *cyaA* gene produces more AC-Hly but not more FHA, it is possible that the excess of AC-Hly is not able to interact efficiently with host cells. Further studies are required to determine whether duplication of *cyaA* gene as well as an increase in production of AC-Hly in vivo, play a role during human infection. 

Clinical isolates, not producing FHA, show also different transcriptomic behaviour since the expression of *ptx*A, *cyaA* or *prn* genes is significantly increased as compared to clinical isolates producing FHA. We are wondering whether this could be a consequence of different regulation, since in these isolates no sequence difference in the *bvg* operon was found [26]. Precise clinical and molecular analyses of isolates freshly collected from infected humans are, therefore, especially important.

### 2.3. Biological Activities

Bp AC-Hly possesses a cell-invasive adenylate cyclase activity and a haemolysin activity. After secretion in the extracellular milieu, Bp AC-Hly binds calcium, and folds into a stable β-sheet conformation that is required for efficient binding to target host cells [27]. The receptor of Bp AC-Hly on host cells is the complement receptor 3 (CR3 or CD11b/CD18 integrin) [28]. Post-translational acylation enhances binding of Bp AC-Hly to CR3 on the surface of phagocytes [29]. However, with lower efficacy Bp, AC-Hly can also penetrate cells lacking the CR3 receptor [30]. After binding to the host cell, Bp AC-Hly is able to translocate the AC domain. Karst et al., showed that deletion of residues 375 to 485 within Bp AC-Hly totally abrogated the toxin’s ability to increase intracellular cAMP in target cells [31]. These results indicate that, while the calmodulin-dependent adenylate cyclase activity is located between the amino acid residues 1 to 384, the membrane-interacting, translocation-competent domain extends up to residue 489 [31].

Furthermore, Bp AC-Hly is also, after oligomerization, a pore-forming cell, responsible for the haemolysis of sheep or horse erythrocytes but not human erythrocytes when incubated using the Bordet Gengou medium [13]. Both invasive and pore-forming activities depend on the post-translational modifications of AC-Hly [13]. Gray et al., demonstrated that close association of live *B. pertussis* bacteria with host cells and ongoing active synthesis and secretion of Bp AC-Hly, are required for delivery of the toxin into target cells. The bacterial surface-associated toxin was found to be unable to penetrate host cells [32].

### 2.4. Role As a Toxin

The first experiment demonstrating that Bp AC-Hly acts as a toxin was reported by Weiss et al. who showed that Tn5-induced *B. pertussis* mutants, non-producing this enzyme, were non-lethal in a murine model [33]. Both adenylate cyclase and hemolytic activities are required for *B. pertussis* to initiate infection [34]. However, using different *B. pertussis* mutants, it has been recently demonstrated that neither the pore-forming (hemolytic) activity of Bp AC-Hly toxin on CD11b phagocytes nor its capacity to elevate cAMP in CD11b cells is per se required for persistent sub-lethal infection of mouse lungs by *B. pertussis*. These capacities, however, are involved in *B. pertussis* penetration across the epithelial lining, provoking enhanced neutrophil infiltration and inflammatory damage of infected tissue [35]. 

The characterized roles of Bp AC-Hly as a toxin are the following:

(i) Bp AC-Hly is able to induce apoptosis of macrophages in vitro and in vivo [36,37]. Apoptotic death of bronchopulmonary cells in vivo was observed exclusively following intranasal infection with bacteria re-isolated from the lungs of infected mice and not with *B. pertussis* collected after in vitro subculture [24]. Depletion of ATP, as a result of adenylate cyclase activity, might be sufficient for macrophage cytotoxicity, but an unconventional calcium influx mediated by the translocating AC domain may also contribute to cytotoxic effects [38,39]. Bp AC-Hly-deficient mutants were shown to be more efficiently phagocytosed by human neutrophils [40], stressing the importance of Bp AC-Hly in targeting phagocytic cells for *B. pertussis* pathogenesis. However, neutrophil depletion did not enhance infection by Bp AC-Hly-deficient *B. pertussis* mutants in naive mice, but in neutrophil-depleted immune (previously infected) mice; the Bp AC-Hly-deficient mutants are as virulent as the parental strain, indicating that the toxin is important in neutrophil inhibition only in the presence of opsonizing antibodies [41].
(ii) *B. pertussis*, as well as Bp AC-Hly, are not cytotoxic for human tracheal epithelial cells [42]. However, we observed induction of IL-6 production by Bp AC-Hly in human tracheal epithelial cells in vitro [43], which enhances recruitment of neutrophils during infection. This observation correlates with our in vivo experiments using the murine respiratory model [24,44].
(iii) Bp AC-Hly production, as well as that of PRN (probably indirectly via its interaction with AC-Hly), inhibits invasion of *B. pertussis* in human tracheal cells, whereas FHA favors this process [42]. It is possible that *B. pertussis* could have evolved anti-invasive mechanisms such as production of AC-Hly to avoid destruction within tracheal epithelial cells. Martin et al., using *B. pertussis* recombinant AC-Hly, showed that the toxin promotes bacterial internalization into non-phagocytic cells. They hypothesize that this internalization favors the persistence of the bacteria in the host. However, they didn’t (a) use human tracheal cells, (b) verify the survival of the bacteria inside the cells, and (c) discuss the fact that *B. pertussis* was not shown to induce persistent or chronic infection in humans, even in immuno-suppressed patients in contrast to some other *Bordetella* species [45]. Further experiments are, therefore, required to investigate these issues.
(iv) While the action of Bp AC-Hly appears to first inhibit bactericidal functions of host innate immunity, such as neutrophils and macrophages, the toxin is likely to have effects on adaptive immunity as well. Bp AC-Hly might mediate an escape strategy for the bacterium, since it reduces Th1 immunity and increases Th17 responses thought to be responsible for enhanced lung inflammation [24,37,44,46,47,48,49].

All the data presented demonstrate that Bp AC-Hly plays an important role during the disease induced by *B. pertussis*. Most of these data derived from in vitro as well as in vivo studies (mostly using the murine respiratory model) and the relevance of these activities to *B. pertussis* infection in humans and pathogenesis remains to be determined. However, a high amount of AC-Hly has recently been detected in nasopharyngeal washes from diseased infants [50], supporting the case for a role of this toxin in respiratory functions in humans. 

### 2.5. Immunogenicity 

Bp AC-Hly is highly immunogenic. Antibodies are detected in serum of infected non-vaccinated subjects [51,52,53,54]. Since none of the actual pertussis acellular vaccines contain Bp AC-Hly, it was proposed to use Bp AC-Hly as an antigen for diagnosis. However, because of its similarities with AC-Hly produced by *B. parapertussis* and *B. bronchiseptica* (as well as other RTX toxins), the results were non-specific. PT antigen remains the only antigen to be used since it is the only one specific to *B. pertussis* [53,55]. Detection of anti-Bp AC-Hly antibodies is variable after vaccination with pertussis whole-cell vaccines [51,53]. However, detection of the antibody following vaccination may vary according to the pertussis whole-cell vaccine used. 

Also a lack or low levels of anti-AC-Hly antibodies has been detected in the serums of children who did not respond to pertussis whole-cell or pertussis acellular vaccines. This could be linked to the “antigenic sin” phenomenon. More studies in vaccinated and infected subjects (children, but also adults) are necessary in order to analyze these findings obtained on low numbers of children.

Recently, Eby et al. [54] demonstrated that anti AC-Hly antibodies developed by humans following infection with *B. pertussis* consistently neutralize toxin-induced cytotoxicity. They suggest that toxin-neutralization assay can be used to characterize the immunological response to AC-Hly after infection and vaccination [54].

### 2.6. Role as a Protective Antigen

At the end of the 1980s, we showed, using the murine respiratory model, that purified Bp AC-Hly can induce a protective immunity against *B. pertussis* infection [56].

Using recombinant Bp AC-Hly (r-Bp AC-Hly), produced by *E. coli* K-12, it has been demonstrated for the first time that r-Bp AC-Hly is a protective antigen in mice [52]. We showed that the cyaC-mediated modifications of the protein are important to induce a protective activity. The region bearing the modifications may be part of an immuno-dominant epitope or the cyaC-mediated modifications may affect the conformation of the molecule, exposing immuno-dominant epitopes [57]. Surprisingly, we also observed that the protective and hemolytic activities of r-Bp AC-Hly were lower than those of Bp AC-Hly produced by *B. pertussis*, while its invasive activity was higher, suggesting that the PTM of AC-Hly in *B. pertussis* and that in *E. coli* K-12 may differ. These observations correlated with the previous data of Hackett et al. [17]. However, in light of our recent data concerning the PTM of Bp AC-Hly produced by *B. pertussis* [18], we favor the hypothesis that the conformation of both proteins might be different because of the lack of interaction of the r-BpAC-Hly with FHA. 

We further used a set of purified fragments of the r-AC-Hly to localize the protective epitopes on the protein. We showed that the structure of the modification-and-repeat region of r-Bp AC-Hly is critical for its protective activity. Furthermore, specific anti-AC-Hly antibodies present in the serum of *B. pertussis*-infected mice and humans are directed, predominantly, against the last 800 amino acids [52]. These antibodies appeared to recognize conformational epitopes present only in a structure formed by the intact C-terminal half of the toxin. However, we didn’t observe any correlation between the capacity of the fragments to induce both toxin-neutralizing antibodies upon immunization of mice and protective immunity, suggesting that the presence of antibodies neutralizing AC-Hly toxin activity might not be a reliable measure of the induced protection against infection by *B. pertussis* [52].

Recently, Wang and Maynard [58,59] confirmed that the C-terminal domain of the toxin is immuno-dominant and elicits neutralizing antibodies, occluding the receptor-binding site. Using several *B. pertussis* isolates, we didn’t find any polymorphism on this C-terminal domain, carrying protective epitopes and the receptor-binding site for human cells of AC-Hly. This finding is also in favor of the important role of AC-Hly in *B. pertussis* pathogenesis [60]. 

However, another region of the toxin, located between amino acid 380–400 and implicated in the translocation of the adenylate cyclase domain [14] is also important. In fact, a monoclonal antibody, able to protect against *B. pertussis* infection, recognizes an epitope localized in this region [61]. Specific polyclonal anti-Bp-AC-Hly antibodies were also shown to be protective against *B. pertussis* infection [62].

Using the murine respiratory model, another property of the Bp AC-Hly has been characterized. It has been observed that inactive Bp AC-Hly enhances protection induced by other pertussis antigens against *B. pertussis* infection, with adjuvant effects on both Th1 and Th2 immune responses [63,64]. 

## 3. *Bordetella parapertussis* and *B. bronchiseptica* Adenylate Cyclase-Hemolysin Toxins

The three classical *Bordetella* species are often considered as subspecies of one species [1,65]. However, it must be noted that the *B. bronchiseptica* species is much more diverse than the *B. pertussis* and *B. parapertussis* species [1]. Furthermore, despite their similarities, their interactions with the human host are not the same. *B. pertussis* and *B. parapertussis_hu_* are the agents of whooping cough. However, while *B. pertussis* produces specifically PT, two fimbrial proteins FIM2 and FIM3, and its lipopolysaccharide (LPS) does not possess an O antigen, *B. parapertussis* does not produce PT, FIM2 and FIM3, and its LPS possesses an O antigen. *B. bronchiseptica* does not produce PT either, but produces and secretes the *Bordetella* type-III secretion system effector A (BteA), a type-VI secretion system, and possesses an LPS presenting high intra-species structural variations [6,8,66]. These discrepancies in the production of virulence factors may explain the difference in these species’ host range, in their behavior inside their host, as well as transmission and adaptability to their environment [1]. However, the three species produce AC-Hly.

### 3.1. Structure 

The structure of the *cya* locus on the chromosome of *B. parapertussis* (Bpp AC-Hly) and *B. bronchiseptica* (Bbs AC-Hly) is similar to that of *B. pertussis* according to the gene sequence [5,67]. The three proteins possess similar adenylate cyclase and haemolytic activities [34,68]. However, few studies were performed on purified Bpp and Bbs AC-Hly. Recently, we determined the PTM of Bpp AC-Hly produced by the reference strain *B. parapertussis* 12,822 and different recent *B. parapertussis* clinical isolates [18]. All Bpp-AC-Hly harbor two PTM corresponding mostly to palmytoylations on Lysine 994 and myristoylations on Lysine 1017 [18].

### 3.2. Regulation of Expression

The expression of Bpp and Bbs AC-Hly are also under the control of the two-component system BvgAS. As observed for Bp AC-Hly, there is probably another regulation in vivo since an increase of expression of Bpp AC-Hly and Bbs AC-Hly by isolates, freshly isolated from lungs of infected mice, is observed [23,24]. 

### 3.3. Biological Activities

In similarity with Bp AC-Hly, the C-terminal part of the Bpp AC-Hly, located between nucleotide 2569 and nucleotide 5121, i.e., from AA857 to AA1706, is conserved. However, in contrast to Bp AC-Hly, Bpp AC-Hly is not cytotoxic for macrophages [18]. This difference might be due either to the different PTM harboured by the two toxins or to different interactions with FHA, PRN or LPS. It is well known that Bpp LPS contains an O antigen, contrary to Bp LPS [69], and it was previously shown that interactions between LPS and RTX toxins are important [70,71].

In contrast to Bp AC-Hly and Bpp AC-Hly, variability is observed with the Bbs C-terminal *cyaA* gene, i.e., between nucleotide 2569 and nucleotide 5121. We identified 14 alleles among 87 isolates analysed [60] and this list might not be exhaustive. Fourteen of the amino acid changes are located on the CD11b/CD18 binding site and might be linked to the sequence differences of the integrin receptor of the various hosts of *B. bronchiseptica* species. However, the 14 Bbs *cyaA* alleles are not host-specific, since they are identified in isolates of either human or animal origin. 

As previously shown [60,72], several *B. bronchiseptica* isolates do not express the toxin, due to a frameshift or a non-sense mutation in the *cyaA* gene or a deletion of the entire *cyaA* gene. In the case of the deletion of the gene, we observed, as per Buboltz et al. [73], that the *cya* operon is replaced by the *ptp* operon. 

Absence of production of an AC-Hly product was observed only in *B. bronchiseptica* isolates independent of their human or animal origin, and this might be at the origin of some peculiarity of the disease induced by this species. We previously showed that expression of the toxin inhibits the invasive properties of *B. pertussis* in epithelial cells [42], and all *B. pertussis* isolates presently express it. As a corollary, no chronic infection has been described to date in humans, even in immuno-compromised subjects, following infection with *B. pertussis*. In contrast, it is known that *B. bronchiseptica* is able to induce chronic infection in mammals [1,72,74], mainly in elderly or immune-compromised patients. Therefore, it is likely that persistence of the infection is favored by the absence of expression of AC-Hly, although the role of this toxin could also be compensated for by expression of another factor, as suggested by Buboltz et al. [73]. It is possible to hypothesize that its expression would be detrimental at some stages of the infectious cycle of *B. bronchiseptica* during chronic infection, although necessary for initiating the infection.

Using the murine model of respiratory infection, it has been shown that both humoral- and cellular-mediated immunities play an important role in the elimination of *B. bronchiseptica* infection [75]. Furthermore, we observed that, after *B. bronchiseptica* infection, a rapid T-cell response is generated to Bbs AC-Hly which is accompanied by an early synthesis of specific AC-Hly antibodies. The importance of Bbs AC-Hly was also confirmed using a porcine model [76]. These results support our previous data showing that *Bordetella* AC-Hly plays an important role in *Bordetella* pathogenesis.

In the last few years, *B. pertussis* and *B. bronchiseptica* have been shown to be able to form biofilms that may play a role in the pathogenesis of these organisms [77,78]. It was observed that a *B. bronchiseptica* mutant lacking AC-Hly produced more biofilm than the parental strain. Furthermore, exogenous AC-Hly inhibits biofilm formation and the N-terminal catalytic domain (AC domain) of the toxin is necessary and sufficient for this inhibitory effect. The AC domain might interact with the C-terminal segment of FHA [78]. 

### 3.4. Role As a Protective Antigen

In similarity with Bp AC-Hly, Bpp AC-Hly and Bbs AC-Hly induce a protective immunity against *B. parapertussis* and *B. bronchiseptica* infections, respectively [68,79]. Since *B. parapertussis* and *B. bronchiseptica* express virulence factors, such as FHA, PRN or AC-Hly, that possess a very strong degree of homology with *B. pertussis* virulence factors, one could expect that an acellular preparation containing these *B. pertussis* antigens would protect against *B. parapertussis* and *B. bronchiseptica* infections. Using purified virulence factors, we demonstrated that it is not the case [68,79], suggesting that the ability of protective antigens to stimulate an effective immune response is species specific. 

The different PTM harbored by Bp AC-Hly and Bpp AC-Hly might be at the origin of the specificity of protection [18]. The other hypothesis might be a different conformation following interactions with other bacterial factors produced by *B. pertussis* and *B. parapertussis*, such as FHA, PRN or LPS, known to be different between the two species [5,69,80,81]. 

## 4. Conclusions

By contrast with other *Bordetella* adhesins or toxins, AC-Hly is one of the few virulence factors that is produced by the three classical pathogenic *Bordetella* species. All the data presented in this review demonstrate that AC-Hly plays a critical role during the disease induced by the classical *Bordetella* species. Furthermore, to the best of our knowledge, no, or very low, polymorphism of the AC-Hly toxin produced by the two human species is observed [18].

The adjuvant and immunomodulatory properties of the inactive Bp AC-Hly also suggest that it has potential as a vaccine component through enhancement of Th1-oriented cell-mediated immune responses.

For all these reasons, many experts in the field propose to include Bp AC-Hly in pertussis acellular vaccines. However, there are still several issues that need to be solved:

(i) *Can genetically inactivated Bp AC-Hly produced by B. pertussis be used?* A *B. pertussis* mutant carrying double mutations in the *cyaA* gene could be used [82]. This double mutant produces a toxoid devoid of both adenylate cyclase and pore-forming activities that may be a suitable antigen for inclusion in a next generation of acellular pertussis vaccines [82]. However, it is well known that the amount of AC-Hly produced by *B. pertussis* is very low. Moreover, Bp AC-Hly is a hydrophobic protein that aggregates or is degraded during purification and needs urea to be solubilized [14,83];
(ii) *Can the genetically detoxified r-Bp AC-Hly, produced by E. coli K-12, be used?* It has been demonstrated that large-scale production of detoxified r-Bp AC-Hly is now possible and clinical cGMP batches have been produced and tested as safe in Phase I clinical trials of T-cell vaccines developed for cancer immunotherapy [83]. However, it was previously shown that the protective activity of Bp AC-Hly and r-Bp AC-Hly were different. This was hypothesized to be due to the different PTM of both proteins [17]. However, we recently showed that this might not be the case [18]. We favor the hypothesis that the difference might be due to the interactions with FHA, PRN and LPS for the final conformation of Bp AC-Hly [19,20,70,71]. Additional investigations are, therefore, requested to test the detoxified r-Bp AC-Hly as an antigen;
(iii) *Can the recombinant monomeric polypeptide which has been recently isolated* [14] *be used?* First, it is necessary to demonstrate that a detoxified monomer induces a protective immunity. Moreover, the production of a high amount of monomeric polypeptide remains to be solved;
(iv) *Can the C-terminal fragment of the toxin produced by E. coli K-12 (and bearing the PTM) be used?* It needs to be confirmed that this recombinant fragment, which induces the synthesis of neutralizing antibodies, also induces a protective immunity, at least in the murine model [58,59]. In fact, we showed, using several different fragments of the r-Bp AC-Hly, that there is no correlation between induction of the neutralizing antibody and induction of a protective immunity [52];
(v) *Can Outer Membrane Vesicule (OMV) produced by B. pertussis be used?* Bacterial OMV naturally contain bacterial surface antigens. The composition of the pertussis OMV was characterized as containing >200 protein components, including the virulence factors PT, PRN, FHA, FIM and AC-Hly. [84,85]. The production of Bp OMV has been developed: (a) the interactions between virulence factors and LPS are preserved; (b) they are immunogenic; (c) they induce an immunity close to that induced after natural infection; (d) they are less reactogenic than pertussis whole-cell vaccines; and (e) induce a protective immunity [84,85]. Furthermore, they contain more antigens than pertussis acellular vaccines and so are less susceptible to inducing the phenomenon of “antigenic sin” [53]. Detoxified Bp OMV can be developed. However, this development will also need further research;
(vi) *Can a mixture of B. pertussis and B. parapertussis OMVs be used?* As shown in France, the circulation of *B. parapertussis* has been slowly increasing for a decade [86]. The increase can be due either to the use of pertussis acellular vaccine or to the evolution of the *Bordetella* species [1,87]. The circulation of *B. parapertussis* has also now been detected in the USA, a country using an acellular pertussis vaccine, but also in Pakistan, a country using a whole-cell pertussis vaccine [3,88]. This means that it could be advantageous to use a vaccine containing both detoxified Bp OMV and Bpp OMV, although it has been shown that Bpp OMV might induce protection against *B. pertussis* and *B. parapertussis* infection [84].

In conclusion, the different properties of the AC-Hly produced by the classical *Bordetella* species described above indicate clearly that this protein plays an important role during the infection induced by these bacteria. Furthermore, no, or very low, polymorphism of the *cyaA* gene in *B. pertussis* and *B. parapertussis* isolates was observed, underlining the importance of AC-Hly in the virulence of these two species, which are pathogenic for humans. The absence of AC-Hly expression by some *B. bronchiseptica* isolates might be related to the specific properties of *B. bronchiseptica* isolates, in particular, their capacity to induce chronic infections. AC-Hly expression might be essential for *B. bronchiseptica*, but not for its persistence inside the host. The polymorphism associated with diversifying selection in *B. bronchiseptica* species might be due to the fact that this species is able to induce chronic infections in a large number of mammalian species, during which it was previously shown that antigen expression varies [72,74].

The introduction of detoxified Bp AC-Hly or r-Bp AC-Hly in pertussis acellular vaccines is of great interest but still requires extensive and lengthy investigation. In particular, the role of interactions with other *Bordetella* vaccine antigens, such as FHA and PRN, in the final conformation of the toxin must be clarified. The use of detoxified Bp and Bpp OMV is of interest, since these interactions between the different *Bordetella* virulence factors are maintained.
